# Supplementary material for: The interactome of intact mitochondria by cross-linking mass spectrometry provides evidence for coexisting respiratory supercomplexes
Source: Mol Cell Proteomics. 2017 Dec 8;17(2):216–32. doi: 10.1074/mcp.RA117.000470 (PMC5795388; doi:10.1074/mcp.RA117.000470)
Supplement: Supplemental Data [file supp_RA117.000470_133922_0_supp_23978_fzffwf.zip › spectra_annotation/mito_spectra_annotation/397-1-5-1-16-1.pdf]

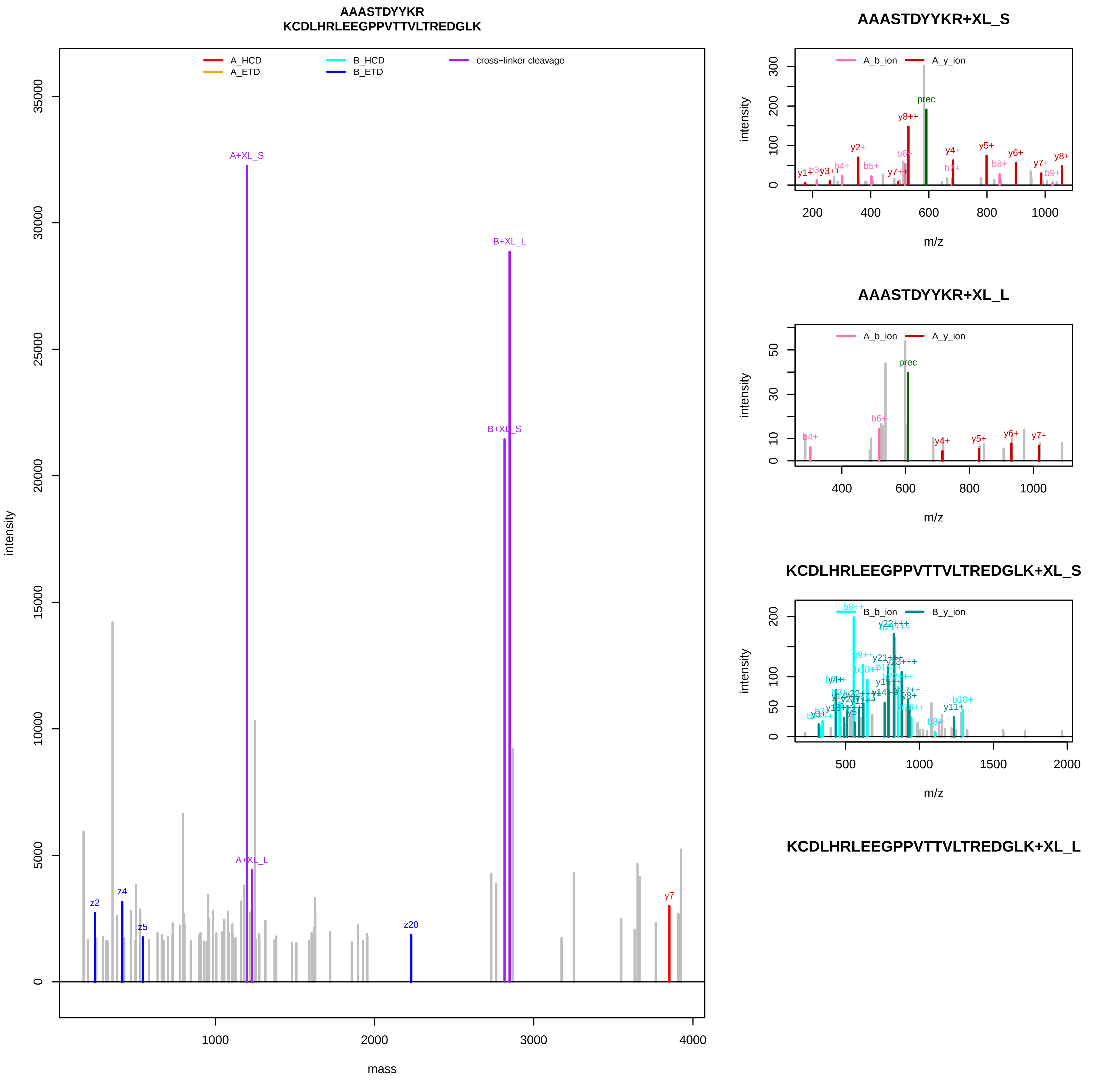
AAASTDYYKR
KCDLHRLEEGPPVTTVLTREDGLK
A_HCD
A_ETD
B_HCD
B_ETD
cross−linker cleavage
A+XL_S
B+XL_L
B+XL_S
A+XL_L
z2
z4
z5
z20
y7
intensity
mass
0
5000
10000
15000
20000
25000
30000
35000
1000
2000
3000
4000
AAASTDYYKR+XL_S
A_b_ion
A_y_ion
prec
y8++
y2+
y4+
y5+
y6+
y7+
y8+
b8+
b9+
b4+
b5+
b6+
b7+
y7++
y1+
y3++
intensity
m/z
AAASTDYYKR+XL_L
A_b_ion
A_y_ion
prec
b6+
b4+
y4+
y5+
y6+
y7+
intensity
m/z
KCDLHRLEEGPPVTTVLTREDGLK+XL_S
B_b_ion
B_y_ion
b8++
y22+++
b9++
y21+++
b10++
y11+
b10+
b8+
intensity
m/z
500
1000
1500
2000
KCDLHRLEEGPPVTTVLTREDGLK+XL_L
